# Maternal diet shapes milk bile acids to regulate neonatal growth through TGR5

**DOI:** 10.1016/j.celrep.2025.116744

**Published:** 2025-12-24

**Authors:** Lufuno Phophi, Haley M. Wilt, Zhengzheng Hu, Rishi Gadikota, Mallory Cadiz, Mikayla S. Manzi, Freddie D. Ortiz Martinez, Sarayu Vanga, Elizabeth G. Chapman, Scott A. Tibbetts, Stephanie M. Karst

**Affiliations:** 1 Department of Molecular Genetics & Microbiology, College of Medicine, University of Florida, Gainesville, FL, USA; 2 These authors contributed equally; 3 Lead contact

## Abstract

Maternal diet is critical in shaping neonatal metabolism and long-term health by governing breast milk composition. Although bile acids are present in breast milk, their functional role in infant development is not well understood. We identify enteromammary trafficking as the primary source of milk bile acids and show that this pool is modifiable by maternal diet. We also find that maternal bile acids regulate infant growth and levels of the growth-promoting hormone insulin-like growth factor 1 (IGF-1). Remarkably, maternal bile acid sequestration completely prevents excess weight gain in offspring nursed by dams on a high-fat diet. Supplementation with a bile acid or an agonist of the bile acid receptor TGR5 restores growth. Furthermore, TGR5-deficient pups phenocopy the maternal sequestration phenotype, supporting the model that maternal milk bile acids activate neonatal TGR5 to promote infant growth. Altogether, these findings reveal milk bile acids as active metabolic signals with potential for nutritional intervention in early-life programming.

## In brief

Phophi et al. show that bile acids in maternal milk act as endocrine signals shaping neonatal growth. Mapping sources and mechanisms, they find diet-dependent remodeling of the milk bile acid pool and TGR5-dependent promotion of pup growth, with reduced IGF-1 linking maternal bile acid levels to infant outcomes.

## Graphical Abstract

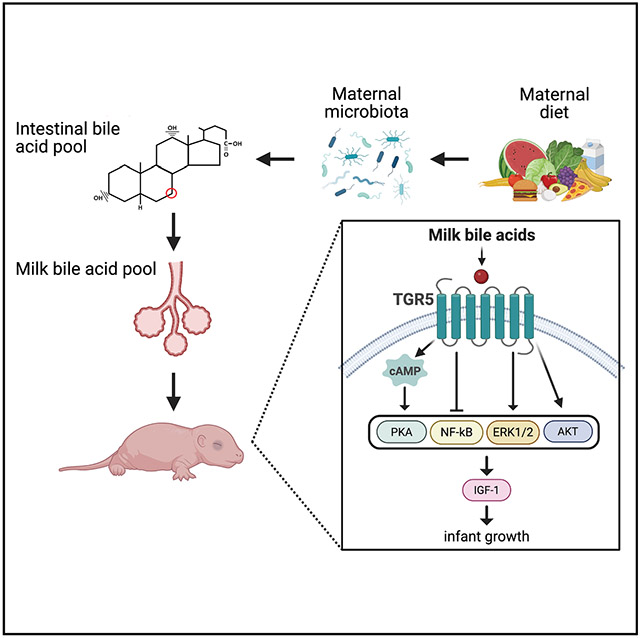

## INTRODUCTION

The world’s leading health organizations unanimously recommend exclusive breastfeeding for the first six months of life. This recommendation reflects the numerous health benefits breastfeeding confers, including optimal nutrient delivery, protection from infections, immune system development, intestinal microbiome maturation, and long-term health implications.^1,2^ Unraveling the molecular underpinnings of these protective effects is critical for developing strategies to optimize infant nutrition and health outcomes.

Mounting evidence indicates that early-life exposures, including breastfeeding, can have lasting consequences on metabolic health. One of the most well-documented outcomes is the programming of body weight. Maternal consumption of a high-fat diet (HFD) during lactation predisposes offspring to obesity, evidenced by increased weight gain during nursing and sustained excess weight post-weaning.^3-5^ These effects have been linked to changes in milk composition, including alterations in hormonal and nutrient content.^3-6^ Conversely, breastfeeding is protective against growth failure in low-resource settings, where it prevents stunting, which involves irreversible physical and cognitive impairments.^7^ Understanding how specific components of milk regulate offspring growth is essential, given the dual global burden of obesity and undernutrition in early life.

Breast milk is a complex and dynamic fluid. While much attention has focused on macronutrients and immunologic factors, small-molecule metabolites are increasingly recognized as key regulators of neonatal physiology, including energy metabolism and immune maturation.^2^ Among these, bile acids have emerged as bioactive milk components with signaling functions.^8-10^ Bile acids are synthesized from cholesterol in the liver, conjugated to either taurine or glycine, and secreted into the intestine to facilitate lipid absorption. They are subsequently modified by gut microbes and recycled via enterohepatic circulation.^11,12^

The presence of microbially derived bile acids in milk suggests that at least a portion of the milk bile acid pool originates from the maternal intestine. Indeed, we previously demonstrated that bile acids can traffic from the maternal intestinal tract to the mammary gland—termed enteromammary trafficking—and that this pathway significantly shapes the milk bile acid profile.^10^ This was supported by findings from both pharmacologic inhibition and genetic disruption. However, reductions in milk bile acids were incomplete, suggesting that additional sources might contribute.

Beyond aiding in digestion, bile acids are potent signaling molecules that influence host metabolism, immunity, and microbial composition.^13,14^ Given their pleiotropic effects, maternally derived bile acids in milk may play important roles in neonatal physiology. Leveraging animal models in which milk bile acid pools are experimentally manipulated, we previously showed that milk bile acids regulate antiviral immunity and protect against intestinal injury.^10^ In this study, we build on that work to investigate whether milk bile acids contribute to regulating infant growth. Specifically, we address the sources of milk bile acids, how maternal diet regulates this pool, and how bile acids in milk govern infant growth.

## RESULTS

### *De novo* synthesis of bile acids in the mammary gland does not occur

**Figure 1.**
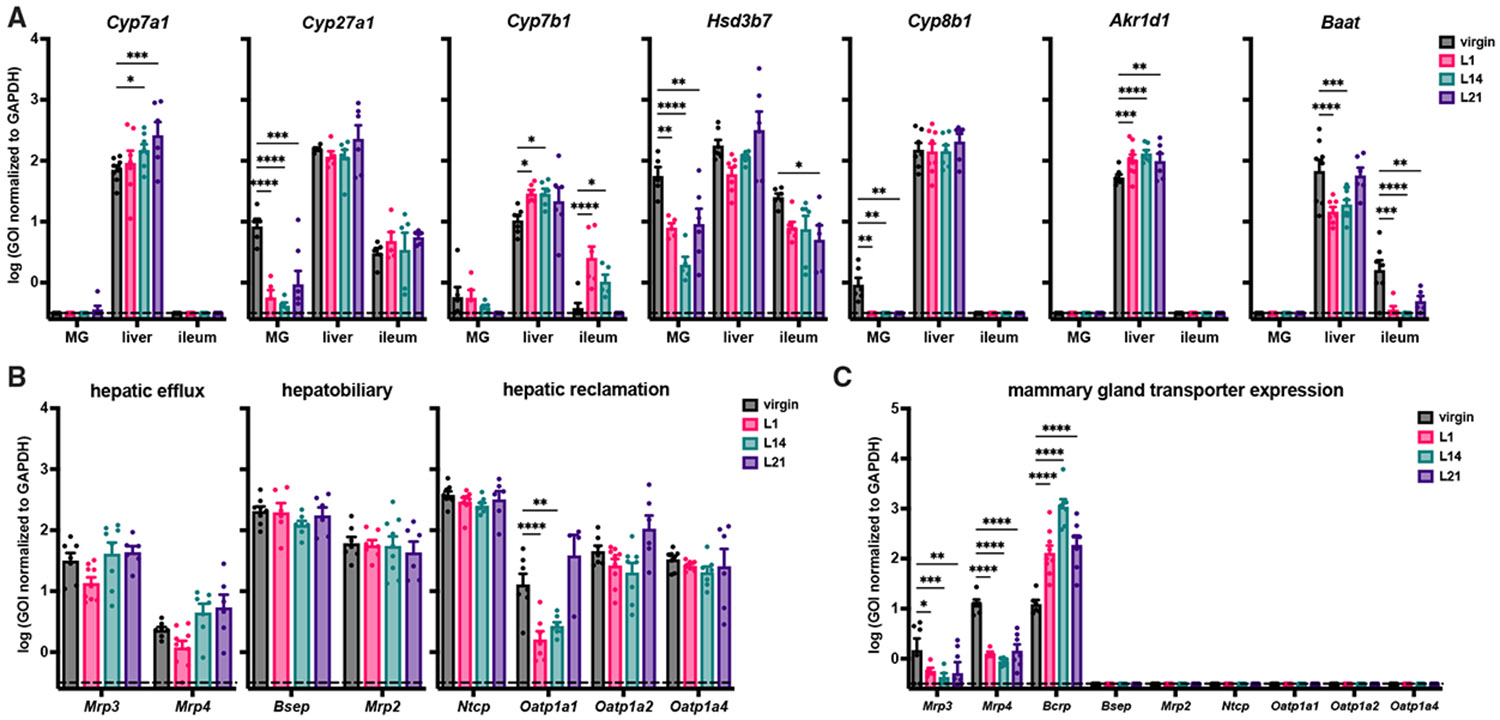
The primary source of milk bile acids is enteromammary trafficking (A) Mammary gland (MG), liver, and ileum tissues were collected from virgin female B6 mice between 8 and 10 weeks old and lactating B6 mice at day 1 of lactation (L1), L14, and L21 for RNA extraction. *Cyp7a1*, *Cyp27a1*, *Cyp7b1*, *Hsd3b7*, *Cyp8b1*, *Akr1d1,* and *Baat* expression levels were determined using quantitative RT-PCR. (B) Livers from virgin female B6 mice between 8 and 10 weeks old and lactating B6 mice at L1, L14, and L21 were harvested for RNA extraction. *Mrp3*, *Mrp4*, *Bsep*, *Mrp2*, *Ntcp*, *Oatp1a1*, *Oatp1a2*, and *Oatp1a4* expression levels were determined using quantitative RT-PCR. (C) MGs from virgin female B6 mice between 8 and 10 weeks old and lactating B6 mice at L1, L14, and L21 were harvested for RNA extraction. *Mrp3*, *Mrp4*, *Bcrp*, *Bsep*, *Mrp2*, *Ntcp*, *Oatp1a1*, *Oatp1a2*, and *Oatp1a4* expression levels were determined using quantitative RT-PCR. Statistical comparisons were performed between levels of gene expression in virgin females and each lactation phase using one-way ANOVA with Dunnett’s multiple comparison test for all images. *p < 0.05, **p < 0.01, and ****p < 0.0001. Data are presented as the mean ± SEM. The precise group sizes are detailed in Table S1.

While our previous study demonstrated that enteromammary trafficking contributes to the milk bile acid pool,^10^ the involvement of additional sources remains plausible. Mammary epithelial cells have an increased capacity to synthesize certain biomolecules during lactation.^15^ Of note, they can synthesize cholesterol,^16,17^ the precursor of bile acids, so it was plausible that *de novo* synthesis in the mammary glands contributes to milk bile acid pools. Consistent with this possibility, there is precedence for extrahepatic bile acid synthesis in the brain^18^ and placenta.^19^ To assess whether mammary glands can produce bile acids during lactation, we analyzed mouse mammary gland tissue from dams at multiple time points during lactation for the expression of key bile acid synthesis genes. Liver and intestinal tissues were analyzed in parallel as positive and negative controls for bile acid synthesis, respectively. Mammary glands collected from virgin, non-lactating female mice were also examined. Bile acid synthesis is a complex process requiring multiple enzymatic reactions that operate in two pathways, the classical and alternative pathways.^11,12^ The initiating and rate-limiting step in the classical pathway is mediated by the enzyme cholesterol 7-alpha-hydroxylase (CYP7A1), which was not expressed in mammary glands collected from lactating dams (Figure 1A), excluding this process from contributing to the milk bile acid pool. Regulation of the alternative bile acid synthesis pathway is more complex, requiring the production of CYP27A1, CYP7B1, hydroxy-delta-5-steroid dehydrogenase, 3 beta- and steroid delta-isomerase 7 (HSD3B7), CYP8B1, aldo-keto reductase family 1 member D1 (AKR1D1), and bile acid CoA:amino acid N-acyltransferase (BAAT). There was minimal expression of most of these genes in intestinal and mammary gland tissue compared to liver tissue (Figure 1A), arguing against the possibility that this pathway operates in the mammary gland to contribute to the milk bile acid pool. Altogether, these results demonstrate that *de novo* bile acid synthesis does not occur in mammary glands.

### Hepatomammary trafficking of bile acids does not contribute to the milk bile acid pool

Given that bile acids are primarily synthesized in the liver, we next considered the possibility that they could traffic directly from the liver to the mammary gland. Hepatic efflux by multidrug resistance-associated protein 3 (MRP3) and MRP4 is generally triggered by increased hepatic bile acid concentrations to prevent toxicity. While *Mrp3* and *Mrp4* expression modestly increased over the course of lactation, it was never significantly elevated compared to basal levels in virgin females (Figure 1B). Likewise, expression of the hepatobiliary transporters bile salt export pump (*Bsep*) and *Mrp2* remained unchanged between lactation and non-lactation conditions (Figure 1B), and sodium taurocholate cotransporting polypeptide (*Ntcp*), the dominant hepatic reclamation transporter, showed no significant changes during lactation (Figure 1B). Interestingly, though, expression of organic anion transporting polypeptide 1a1 (*Oatp1a1*), which contributes to hepatic reclamation of bile acids returning from the intestine, was significantly reduced in lactating dams at day 1 of lactation (L1) and L14 (Figure 1B), possibly contributing to the trafficking of bile acids from the intestinal tract to the mammary gland during lactation. *Oatp1a2* and *Oatp1a4* levels were unchanged during lactation (Figure 1B). Altogether, these findings, together with our prior observations,^10^ support enteromammary trafficking as the primary source of bile acids in maternal milk.

### Expression of bile acid transporters in the mammary gland is limited to MRP3, MRP4, and BCRP

Given that the primary source of milk bile acids is enteromammary trafficking, it is important to understand how these metabolites traverse the bilayered mammary epithelium for secretion into the milk ducts. A previous study reported *Mrp4* expression in mouse mammary tissue,^9^ which we confirmed; however, *Mrp4* levels were significantly reduced during lactation compared to those in virgin females (Figure 1C). *Mrp3* expression followed a similar pattern (Figure 1C). In contrast, and consistent with the findings of Blazquez et al.,^9^ we observed robust expression of the breast cancer resistance protein (BCRP), a broad-specificity efflux transporter known to transport certain modified bile acids. Notably, *Bcrp* expression increased nearly 100-fold by L14 relative to levels in virgin females (Figure 1C). To our knowledge, expression of canonical hepatobiliary and hepatic reclamation transporters (e.g., BSEP and NTCP) has not been previously investigated in the mammary gland. Our analysis revealed that none of these transporters are detectably expressed in mouse mammary tissue (Figure 1C). A prior study suggested that BCRP contributes modestly to bile acid secretion into milk, while MRP4 may serve to restrict bile acid entry into mammary epithelial cells.^9^ Given the absence of other known bile acid transporters, future studies should focus on elucidating specific bile acid substrates transported by BCRP in the lactating mammary gland and investigating whether additional noncanonical bile acid transporters contribute to bile acid trafficking in this tissue.

### Maternal bile acid metabolism regulates offspring growth

**Figure 2.**
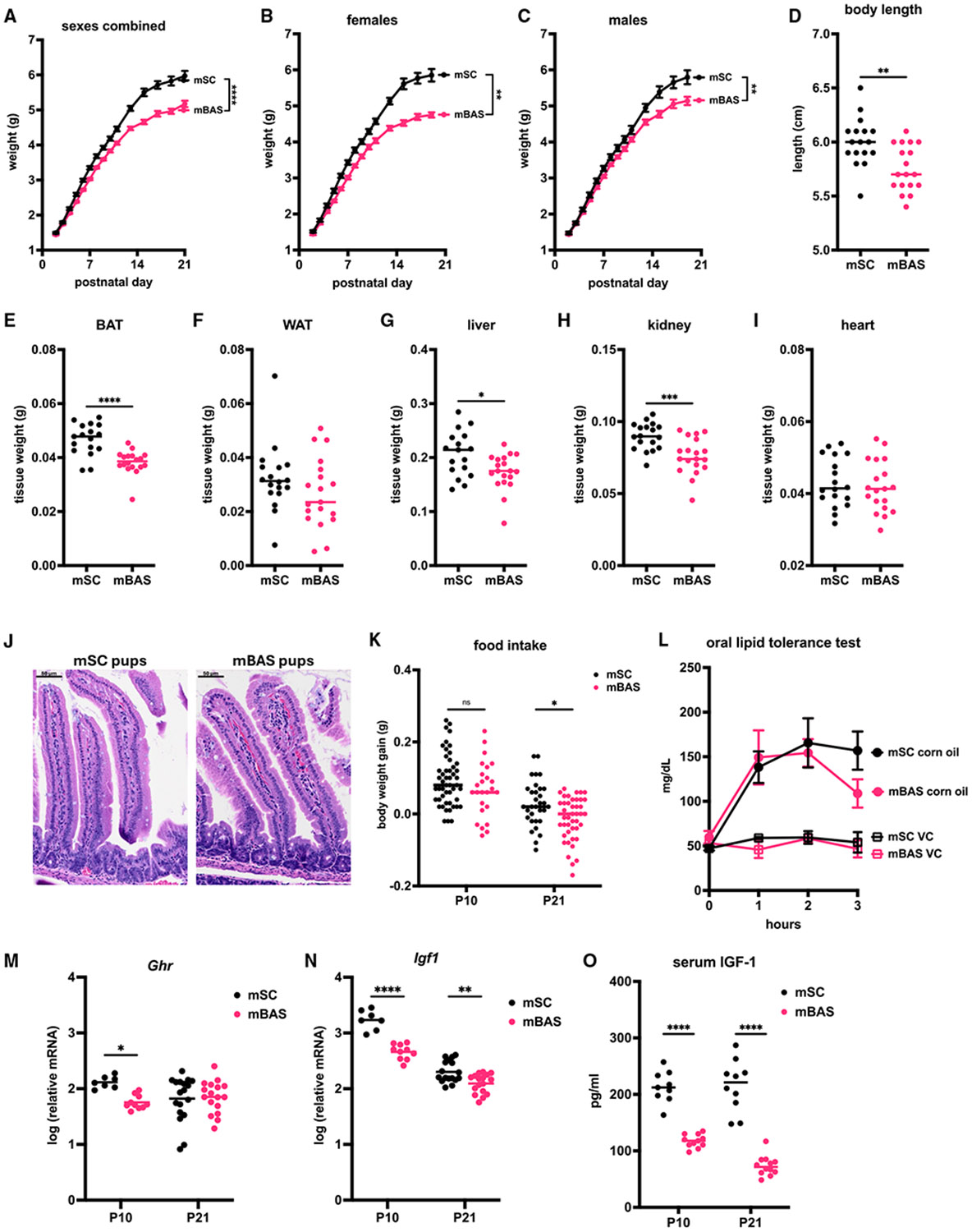
Maternal bile acid metabolism regulates offspring growth and anabolic signaling (A–C) Longitudinal body weight measurements of offspring nursed by dams fed a standard chow (mSC; black) and dams fed an SC supplemented with 5% of the bile acid sequestrant cholestyramine (mBAS; pink) during lactation. Weights for female (B) and male (C) offspring are presented separately. (D–I) Body lengths and weights of the indicated tissues were determined at weaning. (J) Representative hematoxylin and eosin (H&E)-stained distal small intestinal images from P21 pups (scale bar, 50 μm). (K) At the indicated times, pups were separated from dams for 4 h, weighed, reunited with dams for 2 h, and then reweighed. Food intake is reported as the difference in pre- and post-reunification body weights. P21 pups were fasted for 2 h and administered vehicle control (VC) or corn oil (10 μL/g body weight) via oral gavage. Mouse serum was harvested prior to gavage (*T* = 0) and 1, 2, and 3 h post-gavage. (M and N) Liver tissue was harvested from pups nursed by mSC (black) and mBAS (pink) dams at P10 and P21 for RNA extraction. Expression levels of *Ghr* (M) and *Igf1* (N) were determined using quantitative RT-PCR and normalized to the *Gapdh* housekeeping gene. (O) Serum samples were collected from the same groups of mice and assayed for IGF-1 protein by ELISA. Statistical significance was calculated using two-way ANOVA for (A)–(C) and (K)–(O) and Student’s *t* tests for (D)–(I). *p < 0.05, **p < 0.01, and ****p < 0.0001. Data are presented as the mean ± SEM. The precise group sizes are detailed in Table S1.

We previously demonstrated that manipulating the maternal intestinal bile acid pool via oral bile acid sequestrant (BAS) treatment remodels the composition of the milk bile acid pool.^10^ Having confirmed that intestinal bile acids are the main source of the milk pool, we next investigated how these alterations affect neonatal growth. Specifically, pregnant dams were fed standard chow (SC) supplemented with 5% cholestyramine (maternal BAS-treated [mBAS] dams) starting 1–2 days before birth, and infant mice were weighed daily throughout weaning. Newborn mice nursed by mBAS dams were of comparable weight to those nursed by control dams fed standard chow (maternal SC-fed [mSC] dams) at postnatal day (P)2 but displayed reduced weight starting at P6 and weighed 15% less than controls by weaning (Figure 2A). This phenotype was evident in both sexes, although it was more pronounced in females than in males, with 19% and 11% reductions in weight by weaning, respectively (Figures 2B and 2C). These findings reveal that maternal bile acid metabolism governs neonatal weight during the lactation period. Because undernutrition can impair both weight gain and linear growth, we next measured pup body length. Pups nursed by mBAS dams were 5% shorter than mSC-nursed controls at weaning (Figure 2D). To determine whether this early-life growth restriction altered organ development, we evaluated the weights of various tissues. Pups nursed by mBAS dams had significantly lower brown adipose tissue (BAT) (Figure 2E), liver (Figure 2G), and kidney (Figure 2H) weights compared to controls, while white adipose tissue (WAT) (Figure 2F) and heart (Figure 2I) weights were not significantly different. These results suggest selective reductions in tissues involved in digestion, metabolism, thermogenesis, and renal function, with preservation of adipose stores and cardiac mass. A 5% reduction in body length, together with lower body and organ weights, is consistent with mild stunting and suggests impairment of both somatic and skeletal growth.

### Modest intestinal architecture changes in mBAS-nursed pups without marked lipid absorption defects

Because bile acids facilitate dietary fat uptake, we asked whether impaired lipid absorption explains the growth restriction in mBAS-nursed pups. Histology at weaning revealed subtle architectural differences in intestines of mBAS-nursed pups—slightly shorter/blunted villi and a paler, looser lamina propria—but no gross epithelial disruption (Figure 2J). Despite these changes, food intake (Figure 2K) and functional lipid absorption (Figure 2L) were not markedly different between groups. Thus, global growth suppression occurs in the absence of overt malabsorption.

### Anabolic signaling is reduced in pups nursed by mBAS-treated dams

One possible explanation for the observed growth restriction in mBAS-nursed pups is disruption of anabolic signaling pathways that promote tissue growth and energy storage. A major anabolic pathway is the growth hormone (GH) axis. The GH, released from the pituitary gland, binds the GH receptor (GHR) in the liver to stimulate insulin-like growth factor 1 (IGF-1) production, which in turn promotes tissue proliferation. Consistent with maternal bile-acid-mediated regulation of this pathway, mBAS-nursed pups expressed reduced levels of hepatic *Ghr* and *Igf1* at P10 (Figures 2M and 2N). At weaning, mBAS-nursed pups expressed reduced levels of *Igf1* but not *Ghr* (Figures 2M and 2N). Furthermore, serum IGF-1 levels were significantly reduced in mBAS-nursed pups at P10 and weaning (Figure 2O). Together, these findings demonstrate that maternal bile acid metabolism not only shapes early postnatal growth trajectories but also influences anabolic signaling.

### Maternal bile acid metabolism mediates the impact of a HFD on offspring weight gain

**Figure 3.**
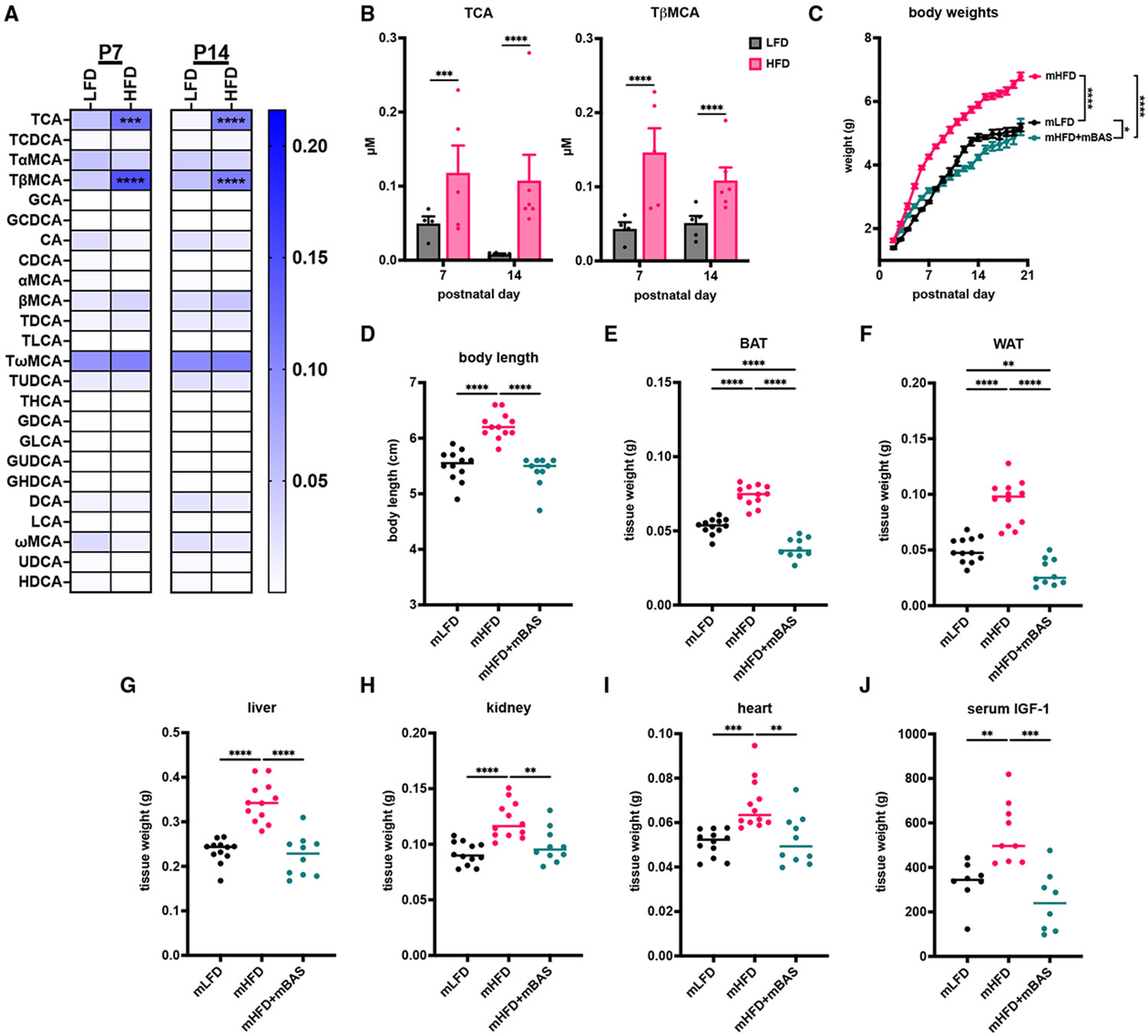
Maternal bile acid metabolism mediates the impact of a high-fat diet on offspring weight gain (A and B) Dam milk samples were collected at P7 and P14 from B6 lactating dams fed LFD or HFD throughout gestation and lactation, and bile acid profiling was performed with ultrahigh-performance liquid chromatography-mass spectrometry (UPLC-MS). (A) A heatmap showing the mean concentrations of individual bile acids. (B) Bar graphs for levels of TCA and TβMCA are shown to visualize within-group variability in diet-regulated bile acids. (C) Pups nursed by dams fed an LFD or HFD beginning 1–2 days before birth and continuing throughout lactation, with BAS treatment initiated 1–2 days before birth (mHFD+mBAS) or without BAS treatment (mLFD and mHFD), were weighed daily during lactation. (D–I) At weaning, body lengths and weights of the indicated tissues were determined. (J) At weaning, serum IGF-1 concentrations (pg/mL) were measured in offspring nursed by mLFD, mHFD, or mHFD+mBAS dams by ELISA. Statistical significance was calculated using two-way ANOVA with Tukey’s multiple comparison test for (A)–(C) and one-way ANOVA with Tukey’s multiple comparison test for (D)–(J). *p < 0.05, **p < 0.01, and ****p < 0.0001. The precise group sizes are detailed in Table S1.

Maternal HFD (mHFD) consumption during lactation predisposes offspring to obesity, as evidenced by greater weight during lactation and persisting into adulthood.^3-5^ This effect has been associated with alterations in the composition of maternal milk.^3-6^ Given the markedly reduced growth of mBAS-nursed pups (Figure 2), we next investigated whether milk bile acids contribute to the increased weight observed in mHFD-nursed pups. First, we mapped the milk bile acid pool in dams fed a control low-fat diet (LFD) or HFD at P7 and P14. Of the 24 bile acids measured (Figure 3A), the levels of taurocholic acid (TCA) and tauro-β-muricholic acid (TβMCA) were significantly elevated in HFD milk compared to LFD milk at both time points (Figures 3A and 3B), representing the first evidence of dietary regulation of milk bile acid composition. To determine the functional consequences of these bile acid changes, we weighed pups nursed by mLFD or mHFD dams throughout lactation, with or without BAS treatment initiated 1–2 days before birth. As expected, mHFD-nursed pups were significantly heavier than those nursed by mLFD dams (Figure 3C). Strikingly, though, BAS supplementation of mHFD dams completely prevented this excess weight gain (Figure 3C). Additionally, mHFD+mBAS-nursed pups had reduced linear growth compared to mHFD-nursed controls at weaning (Figure 3D). When evaluating the weights of various tissues at weaning, mHFD+mBAS-nursed pups had significantly lower BAT, WAT, liver, kidney, and heart weights compared to mHFD-nursed pups (Figures 3E-3I). At weaning, serum IGF-1 concentrations were significantly higher in mHFD-nursed pups compared to those nursed by LFD-fed controls, a phenotype that was reversed by maternal BAS treatment (Figure 3J). Altogether, these results demonstrate that maternal bile acid metabolism contributes to the obesogenic programming induced by the HFD during lactation and that sequestration of intestinal bile acids is sufficient to fully protect nursing offspring from excess weight gain.

### Neonatal TGR5 is required for bile-acid-mediated growth promotion

**Figure 4.**
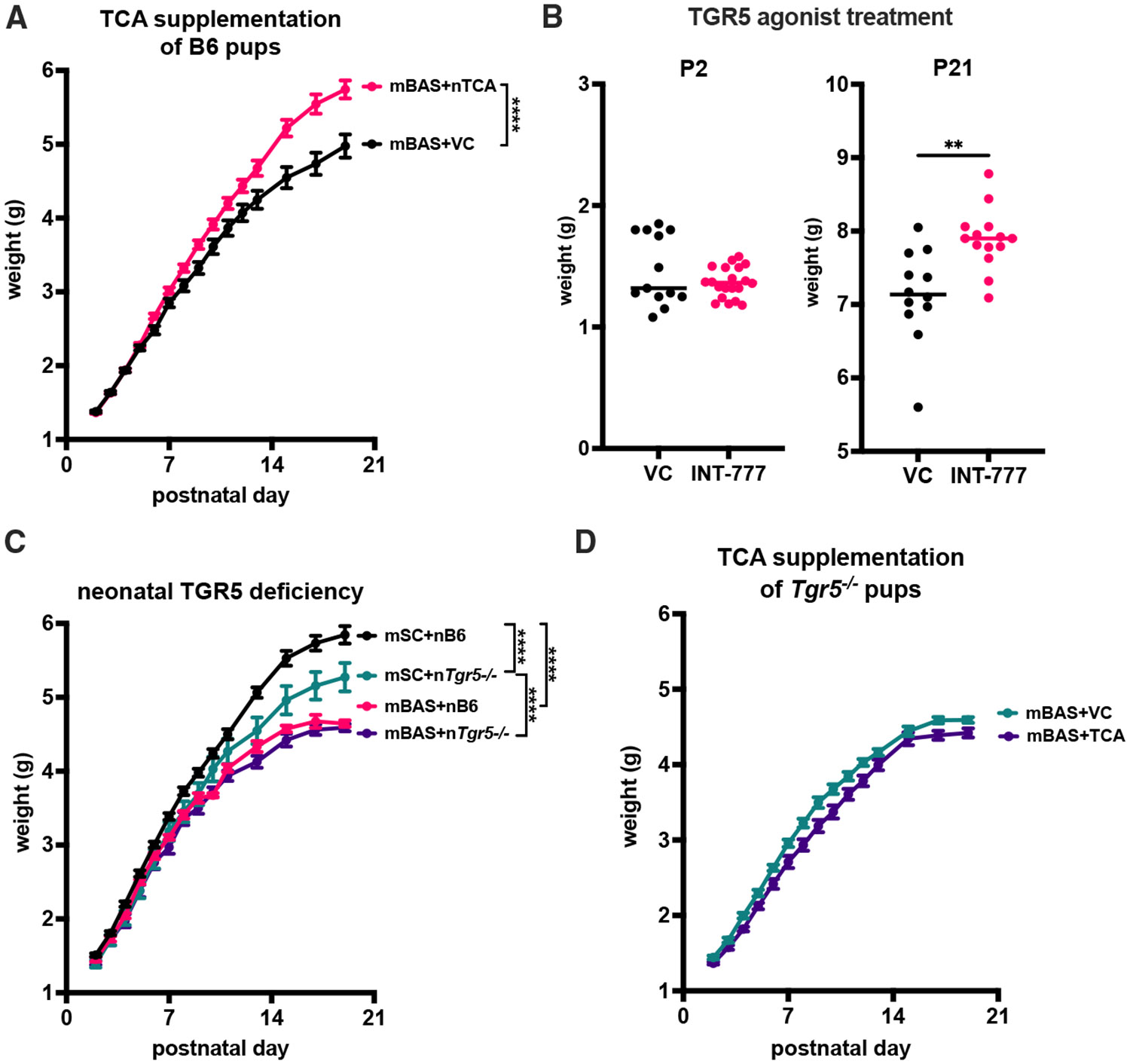
Neonatal TGR5-mediated signaling promotes infant weight gain (A) Neonatal mice nursed by BAS-treated (mBAS) dams were administered 70 μg/g TCA (nTCA) or vehicle control (nVC) daily from P2 to P21, and weights were monitored. Pups nursed by dams fed a standard chow (mSC) and treated with VC were included as controls. (B) Neonatal mice nursed by B6 dams fed a standard chow were administered 60 mg/kg of INT-777 or VC daily from P2 to P21, and weights were determined at P2 and P20. (C) Neonatal B6 (nB6) or n*Tgr5^−/−^* mice were fostered by non-birth B6 dams fed either a standard chow (mSC) or cholestyramine-supplemented chow (mBAS), and the weights were monitored. (D) Neonatal *Tgr5^−/−^* mice nursed by mBAS B6 dams were administered 70 μg/g TCA or VC daily from P2 to P21, and the weights were monitored. Statistical significance was calculated using two-way ANOVA with Tukey’s multiple comparison test for (A), (C), and (D) and unpaired Student’s *t* tests for (B). *p < 0.05, **p < 0.01, and ****p < 0.0001. The precise group sizes are detailed in Table S1.

We next investigated the mechanism by which maternal bile acid metabolism governs infant growth. Given that bile acids are present in dams’ milk^8,9^ and the milk bile acid pool is remodeled by BAS,^10^ maternal bile acids could directly govern newborn growth. Alternatively, mBAS could alter milk composition beyond just bile acids, and these changes could contribute to the observed effects on offspring growth. To distinguish between these possibilities, we supplemented mBAS-nursed B6 pups with TCA, one of the most significantly depleted bile acids in BAS milk,^10^ daily throughout lactation and monitored body weights until weaning. Supporting a direct role for milk bile acids, TCA supplementation partially rescued neonatal weight gain of mBAS-nursed pups (Figure 4A). We next sought to test if bileacid-mediated signaling in newborns contributes to this phenomenon. The G-protein-coupled bile acid receptor TGR5 plays a key role in regulating weight gain in adult mice.^20-22^ To test whether it influences infant growth, we supplemented B6 pups with the TGR5 agonist INT-777^23^ or the vehicle control daily throughout lactation. While initial body weights were comparable, INT-777 supplementation resulted in a 10% increase in body weight by weaning (Figure 4B). To validate the importance of neonatal TGR5 in infant weight gain, we conducted fostering studies in which wild-type B6 or *Tgr5^−/−^* pups were nursed by non-birth B6 dams and weighed until weaning. B6 dams were used to exclude maternal effects of TGR5 deficiency. *Tgr5^−/−^* pups gained 10% less weight than B6 pups by weaning (Figure 4C), supporting a role for TGR5 in promoting optimal postnatal growth. Maternal BAS treatment led to an additional reduction in weight gain in *Tgr5^−/−^* pups (Figure 4C), indicating that while neonatal TGR5 signaling contributes to the mBAS phenotype, it is not solely responsible for it. The absence of an additive effect between mBAS treatment and neonatal TGR5 deficiency suggests that they act within the same pathway (Figure 4C). Importantly, TCA supplementation of *Tgr5^−/−^* pups nursed by mBAS dams failed to promote growth (Figure 4D) as it did in B6 pups (Figure 4A), proving that TCA growth promotion requires neonatal TGR5 signaling. While TCA is a weak TGR5 agonist, it can be deconjugated by bile salt hydrolase (BSH)-positive bacteria to cholic acid (CA) and 7α-dehydroxylated to deoxycholic acid (DCA)—both stronger TGR5 agonists than TCA.^24-26^ Because neonatal mice have minimal 7α-dehydroxylation capacity until near weaning,^27,28^ secondary bile acids remain low throughout most of lactation^10,29^; however, BSH activity is present earlier,^29^ making it plausible that CA, rather than TCA or DCA, contributes to neonatal TGR5 activation during the window when growth effects first emerge. Together, these findings demonstrate that maternal bile acids regulate neonatal growth through TGR5 signaling.

## DISCUSSION

Bile acids were detected in human and rodent breast milk decades ago,^8,9^ yet only recently has their potential role in neonatal physiology been explored.^10^ We previously reported that milk bile acids regulate antiviral immune responses and protect neonatal mice from intestinal injury.^10^ Herein, we extend those findings to demonstrate that milk bile acids act as endocrine-like signals that promote postnatal somatic and skeletal growth through a neonatal TGR5-mediated mechanism. Lowering maternal milk bile acid levels with a sequestrant (mBAS)^10^ depresses neonatal serum IGF-1, reduces body and organ weights, and reduces linear growth without a measurable defect in lipid absorption. Causality is grounded by necessity and sufficiency at the receptor level: the TGR5 agonist INT-777 promotes neonatal growth, and the most abundant milk bile acid, TCA, rescues growth of wild-type pups nursed by mBAS dams but fails to rescue growth of *Tgr5^−/−^* pups. Together, these findings define a milk bile acid-neonatal TGR5 pathway of neonatal growth promotion.

Because bile acids facilitate micellar lipid uptake, impaired absorption could, in principle, explain reduced growth. However, oral lipid tolerance was unchanged, and intestinal histology showed only modest architectural differences without epithelial failure. In contrast, serum IGF-1 was significantly reduced. These observations support the view that, in early life, milk bile acids primarily function as signals that tune endocrine growth pathways rather than functioning as limiting nutrients. Pharmacologic and genetic evidence place neonatal TGR5 as the key receptor through which milk bile acids influence growth, and the concomitant reduction in IGF-1 when maternal bile acid availability is lowered is consistent with crosstalk between milk bile acid-TGR5 signaling and the GH-IGF-1 axis. Nonetheless, we have not demonstrated that TGR5 activation directly upregulates IGF-1. Establishing that causal link remains an important next step. Mechanistically, TGR5 is a G-protein-coupled receptor that raises intracellular cAMP in multiple extrahepatic tissues.^12^ There are multiple ways in which extrahepatic TGR5 activation could modulate hepatic IGF-1 production and stability. First, TGR5 induces type 2 deiodinase in thermogenic tissues, increasing local T3 generation from T4.^30^ Elevated T3 is known to sensitize the hepatic GH pathway.^31-35^ Second, TGR5 activation on myeloid cells dampens pro-inflammatory cytokine expression through cAMP-mediated pathways,^36^ relieving cytokine-induced GH resistance in the liver.^37,38^ Third, enteroendocrine TGR5 in the gut stimulates exocytic release of GLP-1 into circulation, increasing insulin production^39^; insulin, in turn, sensitizes the hepatic GH pathway.^40-43^ Future studies will explore the contributions of each of these pathways to TGR5-mediated promotion of IGF-1 during early life.

mHFD altered the milk bile acid pool and increased offspring weight during lactation. Maternal BAS abolished this HFD-induced excess weight and shifted IGF-1 toward control values, indicating that tuning maternal bile acid availability can interrupt diet-to-infant endocrine programming. Importantly, these effects cut both ways depending on baseline context. Under standard diet conditions, lowering bile acid levels with BAS reduces neonatal growth, a phenotype that could be viewed as undesirable if it pushes infants toward a stunted phenotype. In contrast, in HFD conditions—where milk bile acid levels and IGF-1 are elevated and offspring gain excess weight—lowering bile acid levels prevents overgrowth, a result aligned with obesity prevention goals. Taken together, these dual responses argue that milk bile acid-TGR5 signaling is a bidirectional lever that should be used selectively, not universally. They outline a therapeutic window in which modulating milk bile acid exposure during lactation should be context specific (e.g., mHFD or high bile acid/IGF-1 profiles) and titrated to achieve optimal growth. Practically, this suggests a precision approach, pairing maternal diet and infant growth velocity/IGF-1 as guides for whether to limit milk bile acid levels to curb overgrowth in obesogenic settings.

Upstream mapping of maternal sources of milk bile acids supports enteromammary trafficking as the dominant contributor, with no evidence for *de novo* bile acid synthesis in mammary tissue or hepatomammary contribution, and robust induction of the epithelial exporter BCRP during lactation. We also found that maternal diet shapes the milk bile acid pool: dams fed an HFD exhibit selective increases in TCA and TβMCA at P7 and P14 compared with those fed an LFD, demonstrating dietary regulation of the neonatal bile acid signal delivered in milk. Although bile acids are not dietary in nature, their production is shaped by diet both directly, via hepatic synthesis and enterohepatic feedback responding to nutrient/cholesterol load, and indirectly, through diet-driven shifts in the gut microbiota that modify the bile acid pool. Together, these data define a tractable axis in which maternal enterohepatic bile acid pools, their diet-dependent composition, and mammary export capacity (BCRP) collectively determine neonatal bile acid exposure—and therefore the intensity and composition of receptor signaling in the pup. This framework identifies concrete levers for intervention (modulating maternal bile acid pools, adjusting diet, or targeting mammary export) and provides measurable readouts (milk bile acid species and abundance) to guide context-specific titration of the neonatal bile acid signal.

### Limitations of the study

While this study was designed to illuminate the effects of milk bile acids on neonatal growth during the lactation period, early-life exposures, including lactational nutrition, have lasting effects on offspring metabolic health. Among the best-documented outcomes is programming of growth: mHFD during lactation accelerates offspring weight gain and sustains excess adiposity postweaning, linked to altered milk composition.^3-6^ Thus, future studies will focus on determining whether milk bile acids functioning as early-life endocrine cues have durable metabolic consequences. This includes investigating metabolic health determinants beyond growth, including glucose tolerance, liver function, and adiposity. Moreover, while our findings uncover a direct role for milk bile acids in shaping neonatal growth trajectories—supported by pharmacologic sufficiency (INT-777) and receptor-level necessity (TCA rescue in wild-type but not in *Tgr5^−/−^* pups)—TCA supplementation only partially rescued growth in mBAS-nursed pups. This leaves open the possibility that additional factors influenced by maternal bile acid metabolism contribute to the phenotype. For example, bile acid sequestration is known to remodel the intestinal microbiota,^44-47^ which could in turn alter milk composition and modulate neonatal growth independently of bile acids. Dissecting these indirect routes—e.g., microbiome-driven changes in milk metabolites or immune mediators—will be an important next step.

In sum, milk bile acids act as developmental signals that promote neonatal growth through neonatal TGR5 and suggest crosstalk with the GH-IGF-1 axis. By distinguishing signaling from substrate and identifying a targetable axis in early life, this work provides a mechanistic basis for precisely modulating milk metabolite signaling during lactation—either to prevent overgrowth in obesogenic contexts or to support growth when bile acid levels are low—without altering caloric delivery.

## STAR★METHODS

### EXPERIMENTAL MODEL AND STUDY PARTICIPANT DETAILS

All mice used in this study were bred and housed in animal facilities at the University of Florida. All animal experiments were performed in strict accordance with federal and university guidelines and approved by the Institutional Animal Care and Use Committee at the University of Florida (study numbers 202110473 and 202200000065). The conditions in animal rooms used in this study fall within the standards set by the “Guide for the Care and Use of Laboratory Animals.” C57BL/6J (Jackson no. 000664, referred to as B6) and C57BL/6J-*Gpbar1^−/−^* (referred to as *Tgr5^−/−^*)^49^ mice were used. Neonatal mice in approximately equal sex proportion were used for all experiments. For litter standardization, only litters with 3 or more pups were used. All pups were weighed individually throughout weaning. At indicated times, body lengths were measured from the nose to the tail base (cm). Additionally, brown adipose tissue (BAT), white adipose tissue (WAT), kidney, liver, and heart were removed and weighed.

### METHOD DETAILS

#### RNA extraction and quantitative RT-PCR

RNA was isolated from mammary gland, liver, ileum, and colon tissues of virgin and lactating female B6 mice using TRIzol Reagent (ThermoFisher), treated with Turbo DNase (Invitrogen), and 1 μg RNA was used for cDNA synthesis with the High-Capacity cDNA Reverse Transcription Kit (ThermoFisher). Quantitative PCR (qPCR) was performed using the SYBR Green Master Mix (ThermoFisher) using the primers listed in Table S2. Gene expression was normalized to GAPDH, and all samples were analyzed with technical triplicates.

#### Maternal treatments

For oral bile acid sequestration in lactating dams, dams were fed standard chow (SC) supplemented with 5% cholestyramine (C4650; Sigma-Aldrich) starting 1–2 days before parturition and maintained throughout lactation. Chow was replaced daily. For experiments in which dams were maintained on a high-fat diet (HFD) throughout gestation and lactation, 8 week-old female and male B6 mice were randomly assigned to either an HFD (Research Diets D12492 with 60 kcal% Fat; Brunswick, NJ, USA) or control low-fat diet (LFD) (Research Diets D12450J with 10 kcal% Fat; Brunswick, NJ, USA) at the time of mating. Trio mating was performed to minimize variability due to paternal effects. Pregnancy was confirmed by the detection of a vaginal plug, with the day of detection designated as embryonic day 0.5 (E0.5). After mating, females were individually housed and remained on their assigned diets throughout gestation and lactation. For other experiments, dams were maintained on an HFD throughout lactation only, so groups of pregnant dams maintained on SC throughout gestation were assigned randomly to an HFD or LFD 1–2 days before parturition. For bile acid sequestration in these experiments, females were randomly selected to receive 5% cholestyramine prepared in either HFD or LFD administered 1–2 days before parturition and throughout lactation. Chow was changed daily.

#### Bile acid profiling

For mouse dam milk bile acid profiling, lactating dams were milked on P7 and P14. On the day of milking, litters were separated from dams for 2 h and dams were given 2 international unit (IU)/kg of oxytocin diluted in saline (100 μL total volume) intraperitoneally (i.p.) and anesthetized using isoflurane. Eye lubricant was used for the duration of the procedure. Once anesthetized, milk was expressed from each mammary gland, collected using a micropipette, and frozen. Anesthesia time never exceeded 25 min total time. When the procedure was complete, the dams were monitored to ensure no side effects. 20 μL of each sample was mixed with 80 μL of acetonitrile. After vortexing, sonication for 5 min in an ice-water bath, and centrifugation at 21,000 g for 15 min, 80 μL of the clear supernatant was mixed with 40 μL of the internal standard solution and 880 μL of water. The mixture was loaded onto a reversed-phase solid-phase extraction cartridge (60mg/1mL). After sample loading under a positive pressure, the cartridge was washed with 2 mL of water. Bile acids were eluted with 1 mL of methanol under the positive pressure. The collected fraction was dried in a nitrogen gas evaporator. The residue was reconstituted in 40 μL of 50% acetonitrile. 10 μL aliquots of each fecal and milk sample solutions and each of the calibration solutions were then injected into an Agilent 1290 UHPLC system coupled to an Agilent 6495B QQQ mass spectrometer. The MS instrument was operated in the multiple-reaction monitoring (MRM) mode with negative ion detection. A Waters C18 column (2.1*150 mm, 1.7 μm) was used for LC separation and the mobile phase was 0.01% formic acid in water and in acetonitrile for binary-solvent gradient elution. A mixed solution of all the targeted bile acids at 10 μM of each compound was prepared in an internal standard solution of 14 deuterium-labeled bile acids in 50% acetonitrile. This solution was further diluted step by step to have 10 calibration solutions. Linear-regression calibration curves of individual bile acids were constructed with the data acquired from injection of the serially diluted calibration solutions. Concentrations of bile acids detected in the samples were calculated by interpolating the calibration curves of individual bile acids with the analyte-to-internal standard peak area ratios measured form injection of the sample solutions. Bile acid profiling was performed by Creative Proteomics.

#### Neonatal supplementations

For bile acid supplementations, neonatal mice nursed by mBAS dams were supplemented with 70 μg/g dose of TCA (Cayman Chemical) in PBS daily beginning at P2 using intragastric (i.g.) inoculation, as previously published.^48^ When mice reached P9, oral gavage was used for inoculations instead since the milk spot was no longer visible.^50^ Controls pups nursed by mSC and mBAS dams were given PBS in the same manner as a vehicle control (VC). For TGR5 agonist treatment, neonatal mice nursed by wild-type B6 dams were administered 60 mg/kg of INT-777 (MedChem Express) in corn oil in 20 μL via i.g inoculation between P2 and P9 and then using oral gavage through P21, or corn oil as VC.

#### Food intake and lipid absorption measurements

At the indicated times, pups were fasted for 4 h and weighed. They were then reunited with dams and allowed to suckle for 2 h before being reweighed. Food intake is reported as the difference in pup weight between pre- and post-reunification time points. For oral lipid tolerance tests, pups were fasted for 2 h and then treated with 10 μl/g of body weight PBS or corn oil. Serum was collected at 0, 1, 2, and 3 h and triglycerides were measured using a triglyceride colorimetric assay kit (Cayman Chemical).

#### Serum IGF-1 analysis

IGF-1 levels were determined using the Mouse IGF-1 ELISA Kit (R&D Systems, MG100) according to the manufacturer’s guidelines.

### QUANTIFICATION AND STATISTICAL ANALYSIS

All data were analyzed with GraphPad Prism software. *p* values were determined using unpaired Student’s t-tests, one-way ANOVA, or two-way ANOVA with corrections for multiple comparisons, as specified in each figure legend. *p* values less than 0.05 are indicated by one asterisk, *p* values less than 0.01 are indicated by two asterisks, *p* values less than 0.001 are indicated by three asterisks, and *p* values less than 0.0001 are indicated by four asterisks. Precise animal group sizes are detailed in Table S1. Error bars denote standard errors of mean in all figures

## Supplementary Material

Supplementary table 2

Supplementary table 1

Supplemental information can be found online at https://doi.org/10.1016/j.celrep.2025.116744.

## Highlights

- Enteromammary trafficking is the primary source of milk bile acids during lactation
- Maternal diet remodels milk bile acids during lactation
- Milk bile acids promote neonatal growth via TGR5 activation
- Milk bile acids increase levels of the growth-promoting hormone IGF-1
